# Does a combination treatment of repetitive transcranial magnetic stimulation and occupational therapy improve upper limb muscle paralysis equally in patients with chronic stroke caused by cerebral hemorrhage and infarction? A retrospective cohort study

**DOI:** 10.1097/MD.0000000000026339

**Published:** 2021-06-18

**Authors:** Hisashi Tatsuno, Toyohiro Hamaguchi, Jinichi Sasanuma, Kiyohito Kakita, Takatsugu Okamoto, Masato Shimizu, Naoki Nakaya, Masahiro Abo

**Affiliations:** a Department of Rehabilitation Medicine, The Jikei University School of Medicine, Tokyo; b Department of Rehabilitation, Graduate School of Health Sciences, Saitama Prefectural University, Saitama; c Shin-Yurigaoka General Hospital, Tokyo; d Kyoto Ohara Memorial Hospital, Kyoto; e Nishi-Hiroshima Rehabilitation Hospital, Hiroshima; f Shimizu Hospital, Tottori, Japan.

**Keywords:** cerebral infarction, Fugl-Meyer assessment, hemorrhage, intracerebral, occupational therapy, repeated transcranial magnetic stimulation, upper extremity

## Abstract

The clinical presentation of stroke is usually more severe in patients with intracerebral hemorrhage (ICH) than in those with cerebral infarction (CI); recovery of stroke-related muscle paralysis is influenced and limited by the type of stroke. To date, many patients have been treated by neurorehabilitation; however, the changes in the recovery of motor paralysis depending on the type of stroke, ICH or CI, have not been established. This study aimed to determine this difference in improvement of upper extremity paralysis using 2-week in-hospital NovEl intervention Using Repetitive transcranial magnetic stimulation combined with Occupational therapy (NEURO).

We scrutinized the medical records of all patients with poststroke (ICH or CI) upper extremity muscle paralysis using Fugl-Meyer assessments (FMAs) who had been admitted to 6 hospitals between March 2010 and December 2018 for rehabilitation treatment. This was a multiinstitutional, open-label, retrospective cohort study without control patients. We evaluated the effects of NEURO on patients with CI and ICH by dividing them into 2 groups according to the type of stroke, after adjustment for age, sex, dominant hand, affected hand side, time since stroke, and prediction of recovery capacity in the upper extremity.

The study included 1716 (CI [n = 876] and ICH [n = 840]) patients who had undergone at least 2 FMAs and had experienced stroke at least 6 months before. The type of stroke had no effect on the outcomes (changes in the FMA-upper extremity score, *F*_[4,14.0]_ = 2.05, *P* = .09, partial η^2^ = 0.01). Patients from all 5 groups equally benefited from the treatment (improvement in FMA scores) according to the sensitivity analysis-stratified analysis (*F* = 0.08 to 1.94, *P* > .16, partial η^2^ < 0.001).

We conclude that NEURO can be recommended for chronic stroke patients irrespective of the type of stroke.

## 1 Introduction

The clinical presentation of stroke is generally more severe in patients with intracerebral hemorrhage (ICH) than in those with cerebral infarction (CI). ICH is associated with significant mortality rates, especially in the first 3 months of the poststroke period.^[1]^ In addition, the extent of motor paralysis and sensory deficits at recovery are different between infarction and hemorrhage.^[2]^ Stroke recovery is focused on the motor deficit, in addition to the size and location of the brain lesion.^[3]^

Poststroke motor paralysis affects the activities of daily living (ADL), including personal and social activities, and is associated with poor quality of life.^[4]^ In particular, the ADL of stroke patients is affected by motor paralysis of the upper limbs. Therefore, several institutions have included neurorehabilitation as part of the overall clinical management of these patients.^[5]^ Neuromodulation and changes in brain blood flow are realized following repeated transcranial magnetic stimulation (rTMS) for the treatment of mild or moderate upper limb paralysis in the chronic phase, as demonstrated by studies using single-photon emission computed tomography^[6]^ and functional magnetic resonance imaging.^[7]^ Decreasing excitability of the cerebral cortex is associated with a decrease in excitability of the anterior horn cells on the paralyzed side,^[8,9]^ thus improving the spasticity of the muscle tones.^[10,11]^ Therefore, it has been reported that treatment with rTMS improves patient paralysis and increases patient satisfaction.^[11]^

One of the methods used in neurorehabilitation is the NovEl intervention Using Repetitive transcranial magnetic stimulation combined with Occupational therapy (NEURO); rTMS for the regulation of interhemispheric inhibition imbalance is an additional therapy for poststroke motor function.^[12–14]^ NEURO modulates the excitability of the motor cortex by rTMS; it aims at stimulating peripheral muscle movement and active exercises for the paralyzed muscles of the upper limbs.^[15]^ The typical rTMS frequency of 10 Hz has a promoting effect on brain activation, whereas 1 Hz has an inhibitory effect. The stimulation intensity of rTMS is 1.5 to 2.0 Tesla, and the stimulation depth is 1.5 to 3.0 cm.^[16]^ To reduce hyperexcitability of the intact motor cortex that occurs in moderate to mild upper limb paralysis in the chronic phase, low-frequency rTMS is performed on the intact motor cortex,^[17,18]^ the frequent rTMS is applied to the motor cortex on the affected side to enhance motor function in the acute phase.^[19,20]^ Intensive exercise represented by constraint-induced movement therapy is known to improve upper limb paralysis in the chronic phase.^[21]^ However, a previous report indicates that low-frequency rTMS before and after training is more effective in improving upper limb function compared to constraint-induced movement therapy.^[22]^

A clinical question is whether NEURO has differing effects in ICH and CI cases. Ideally, physicians should inform patients about the effectiveness of NEURO in motor paralysis caused by ICH or CI. ICH and CI are clinically categorized in the same group of disorders, and considering that patients undergoing NEURO are usually in the chronic phase, whether motor paralysis is due to ICH or CI is of little concern. The efficacy of the NEURO program has been confirmed through several randomized control trials,^[18,23]^ and to date, many patients have been treated with NEURO. However, there has been no comparison between its efficacy and outcomes (recovery of muscle paralysis) in patients with ICH and in those with CI. In this study, the main hypothesis tested was that the effectiveness of NEURO in terms of recovery of upper extremity motor paralysis would be equivalent in patients with ICH and CI.

## 2 Materials and methods

### 2.1 Ethics and patient consent

This retrospective and longitudinal study was approved by the Human Ethics Committee of Tokyo Jikei University School of Medicine (20-268-5558). Each patient provided an opt-out signed consent form before the start of NEURO, agreeing to have their treatment data analyzed, including in any retrospective studies conducted after treatment. Furthermore, the study was conducted according to the principles of the Declaration of Helsinki.

### 2.2 Participants

This is a multi-institutional open-label study without control patients. In January 2019, we scrutinized the medical records of all patients with poststroke muscle paralysis who had been admitted to 6 participating institutions (Jikei University Hospital, Jikei Third Hospital, Tokyo General Hospital, Kyoto Ohara Memorial Hospital, Nishi-Hiroshima Rehabilitation Hospital, Shimizu Hospital) between March 2010 and December 2018 for NEURO. The diagnoses of ICH and CI were established for clinical care, and we obtained diagnosis data from the medical records. We used the following inclusion criteria^[16]^ for patient recruitment based on the rTMS guidelines: upper limb hemiparesis associated with CI or ICH, age > 20 years, ≥6 months since the stroke, history of a single stroke only (no bilateral cerebrovascular lesions), and data available on Fugl-Meyer assessment (FMA) test before and after NEURO. The fifth criterion was included to assess the effects of NEURO. We excluded patients based on the following criteria: cognitive deficits (a mini-mental state examination score ≥26), active physical or mental illness requiring medical management, history of convulsions for ≥1 year, intracranial metal clips or intracardiac pacemaker, chemodenervation of the affected upper limb with phenol or botulinum toxin, and subarachnoid hemorrhage.

### 2.3 NEURO in combination with occupational therapy

All patients were hospitalized for 15 days to receive rTMS^[24]^ and occupational therapy (OT).^[8]^ During hospitalization, each patient received a 40-minute low-frequency rTMS therapy and a 60-minutes OT twice daily, excluding the admission/discharge days and Sundays. A MagPro R30 stimulator (MagVenture Company, Farum, Denmark) with 70-mm loop diameter figure-8 coils was used for rTMS. In each session, 2400 stimuli of 1 Hz each were applied for 40 minutes to the contralesional hemisphere over the primary motor area. The stimulation intensity was set at 90% of the resting motor threshold of the first dorsal interosseous muscle, which was defined as the lowest stimulation intensity that induced motor-evoked potentials in the interosseous muscle. Throughout the rTMS sessions, all patients were monitored carefully by the attending physiatrist.

OT included individual training sessions. The main goal of the OT sessions was to divert patients from focusing on functional training and encourage them to use their affected upper limbs again in daily activities. The treatment strategy included performing daily physical activities (i.e., eating) that included repetitive flexion and extension movements of the arm; performing individualized functional training tasks, such as washing hands and grasping small items with the paralyzed fingers, which improved upper limb movements; training the gross motor, fine motor, and multitasking skills; clearly demonstrating the position of the upper limbs to draw attention to this position during training; staging interventions; performing ADLs and unsupervised training tasks that could be continued after discharge; and providing action feedback by passive intervention with verbal instructions.

Patients were treated by OT and physiotherapy for 20 minutes per session, with a maximum of 6 sessions per day. For NEURO of the upper limbs only, patients received OT for all 6 sessions. Depending on the patients’ demands, physiotherapy and OT were given in 2 to 3 sessions, respectively. This allocation was decided through discussions between a physician and the patient.

### 2.4 Samples for analysis

Based on repeated measures analysis of variance (F tests) with power (1-β) of .95, α of .05, critical F of 2.22, 2 groups and 2-time courses, and 6 variances explained by special effects, the estimated minimum sample size was 230 patients (based on G∗Power 3.1 software, Heinrich-Heine-Universität Düsseldorf, Dusseldorf, Germany). Therefore, we planned to recruit at least 460 stroke patients treated with NEURO. We analyzed the data from 876 patients with ICH and 840 patients with CI to determine the effects of NEURO on upper limb muscle function.

### 2.5 Outcomes

To determine the differences between the 2 groups of patients, we compared the FMA scores (pre- and post-NEURO), age, sex, dominant hand, and time to recover the muscle function after stroke onset. The primary outcomes were the FMA scores.

### 2.6 Clinical evaluation of motor function

The motor function of the affected upper limb was evaluated on the day of admission and the day of discharge using FMA. FMA is a performance-based quantitative measure composed of 33 items that evaluates the upper extremity motor function. Each item is rated on a 3-point ordinal scale (0 = cannot perform, 1 = can perform partially, and 2 = can perform fully), with a maximum score of 66 points.^[25]^ The severity of paralysis, according to the FMA score for upper extremity function (FMA-UE) observed previously, was classified as follows: no (<23), poor (≤23–<32), limited (≤32–<42), notable (≤ 42–<53), or full (≤53) upper limb capacity at 6 months poststroke.^[26]^ The minimal clinically important difference identified in the FMA-UE in a population of stroke patients is 4 to 10 points in the acute or subacute phase^[27]^ and 5 points or less in the chronic phase.^[5]^

### 2.7 Statistical analysis

This study was conducted without control patients. Therefore, we performed a prebasal evaluation to verify the pretreatment stability of the effectiveness endpoints using a within-subject time-series design to compare NEURO outcomes in patients with ICH and CI. Two analyses were performed with and without imputing data; the imputed data of FMA-UE (pre- or post-treatment) scores were used in the main analysis.^[28]^ In the analysis with imputed data, a linear regression model was used to determine the changes in patients’ performance with a data analytic (FMA score) and the missing data strategies over time.^[29]^ For example, when one FMA score was missing, the slope of the change was calculated from a linear regression equation of all completed data, and a score of the patient with missing data was estimated and imputed for analysis; vice versa, patients with missing data were removed.

**Figure 1:**
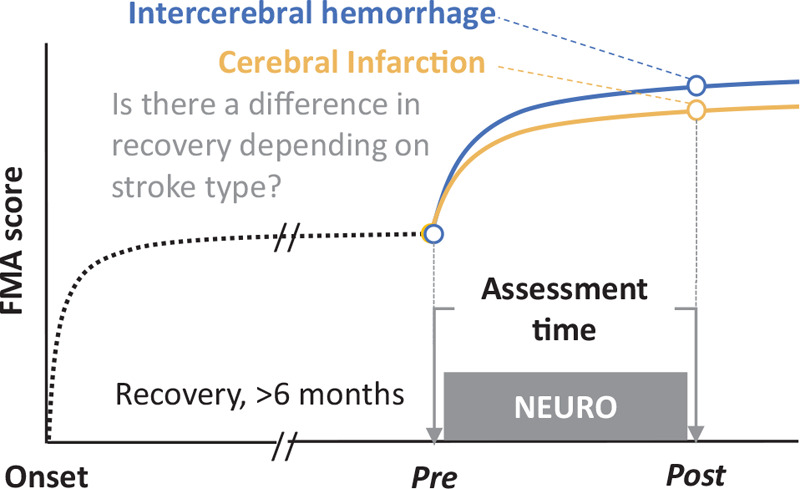
Study design. The Fugl-Meyer assessment (FMA) score was recorded before and after a 2-week NovEl intervention Using Repetitive transcranial magnetic stimulation and Occupational therapy (NEURO) treatment in chronic stroke patients with cerebral infarction and intracerebral hemorrhage. The data obtained pre- and post-NEURO were verified and statistically compared.

First, to determine the differences in the therapeutic effects of NEURO between stroke patients (ICH and CI), repeated measures analysis of variance was performed based on the time required for recovery from upper limb muscle paralysis (changes in FMA scores) and diagnosis (CI or ICH) (Fig. 1). The factors influencing recovery after muscle paralysis,^[30]^ such as age, sex,^[31,32]^ time from stroke onset,^[1]^ affected area of the cerebral hemisphere,^[33,34]^ and handedness were used as covariates for confounder-adjustment estimates. Adjustment for multiplicity was performed using the Bonferroni correction. For detailed verification, the delta FMA score was compared between the ICH and CI groups using the Student *t*-test. Before the analyses, assumption checks were performed using the Levene homogeneity of variances test and the Shapiro-Wilk normality test for the FMA-UE score. Second, sensitivity analysis–stratified analysis was used to evaluate the pretreatment severity (predicted recovery capacity) of upper extremity paralysis. For sensitivity analysis, we divided the pretreatment FMA-UE score into no (<23), poor (≤23−<32), limited (≤32−<42), notable (≤42−<53), or full (≤53) upper extremity capacity.^[26]^ We defined statistical significance as *P* < .05. All analyses were performed using the R software version 3.6.0 (R Foundation for Statistical Computing, Vienna, Austria).

## 3 Results

**Figure 2:**
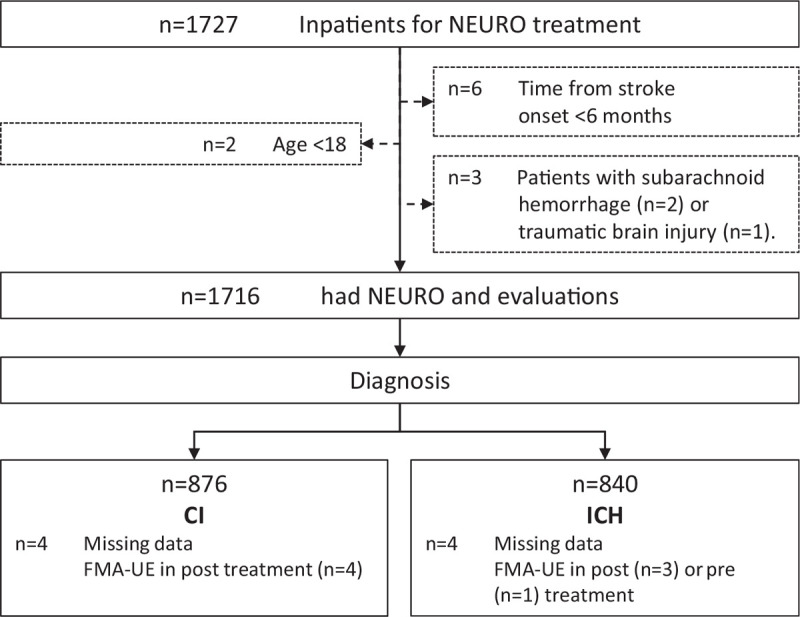
Flow chart of the subject recruitment protocol followed in this study. CI = cerebral infarction, FMA-UE = Fugl-Meyer assessment score for upper extremity function, ICH = intracerebral hemorrhage, NEURO = NovEl intervention Using Repetitive transcranial magnetic stimulation and Occupational therapy.

Figure 2 shows a flow chart of the study design and patient recruitment. The median (25th–75th percentiles) age of the patients was 63 (56–70) years. Table 1 summarizes the clinical characteristics of the patients; the distribution of clinical features was comparable between the 2 groups. A total of 1727 stroke patients received NEURO treatment during the study period. However, 11 patients did not meet the inclusion criteria—2 were younger than 18 years, 6 were <6 months from onset, 2 had subarachnoid hemorrhages, and 1 had brain trauma. There were 840 patients in the ICH group and 876 in the CI group; data were missing for 8 patients. Finally, 1716 patients were analyzed using imputed data. The sample size was adequate in both groups. Right-handed patients accounted for 97% of the total sample population, which was almost equal to the entire sample population. The number of men was about twice that of women in both the groups.

**Table 1:** Baseline characteristics of the patients in the 2 groups.

		All	ICH	CI
N		1716	840 (49%)	876 (51%)
Age, y		63 (55–70)	60 (54–68)	63 (58–72)
Sex (n)	Female	582 (34%)	300 (34%)	282 (34%)
	Male	1134 (66%)	576 (66%)	558 (66%)
Paralysis side (n)	Left	742 (42%)	391 (45%)	351 (42%)
	Right	974 (58%)	485 (55%)	489 (58%)
Dominant hand (n)	Left	97 (6%)	50 (6%)	47 (6%)
	Right	1619 (94%)	826 (94%)	793 (94%)
Time from onset, mo		41 (23–74)	46 (25–79)	37 (22–67)
FMA-UE score (pretreatment)		49 (38–56)	48 (38–56)	50 (39–56)

**Figure 3:**
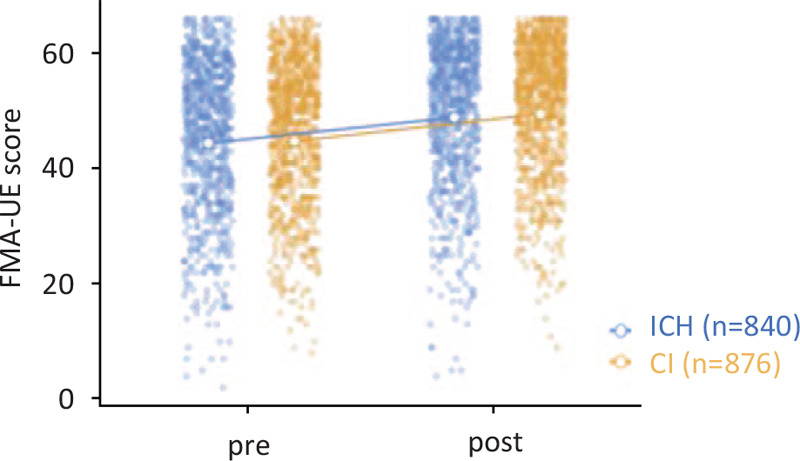
Effects of treatment on Fugl-Meyer assessment (FMA) scores of the upper extremity. Treatment time series had a significant effect on the Fugl-Meyer assessment score for upper extremity function (FMA-UE) scores (*F*_[1,161.749]_ = 23.64, *P* = .0001, partial *η*^2^ = 0.01). There was no significant interaction between diagnosis (cerebral infarction and cerebral hemorrhage), time series (pre- and post-NEURO), and recovery capacity prediction of the FMA-UE (*F*_[4,13.97]_ = 2.05, *P* = .09, partial η^2^ = 0.01). Open circles and error bars denote the estimated marginal mean and standard errors.

The pretreatment stability of the effectiveness endpoints was verified, and no significant difference was found in the pretreatment FMA-UE scores between the diagnoses of ICH and CI (*t* = 1.34, *P* = .18, Cohen *d* = 0.065, Table 2). Figure 3 shows the time-series plots of FMA scores.

**Table 2:** Comparing the pretreatment Fugl-Meyer assessment scores of the upper extremity between 2 types of strokes and verifying the pretreatment stability of the effectiveness endpoints.

						95% Confidence interval		
Time-series	Diagnosis	FMA-UE score	*t*	*P*	Mean difference	Lower	Upper	Cohen *d*
Pre	ICH (n = 840)	48 (38–56)	1.34	.18	0.81	−0.38	1.99	0.065
	CI (n = 876)	50 (39–56)						

Comparing the within-subjects effects, there was no significant interaction between diagnosis (CI and ICH), time series (pre- and post-NEURO), and recovery capacity prediction of the FMA-UE (*F*_[4,14.0]_ = 2.05, *P* = .09, partial η^2^ = 0.01). Treatment time had a significant effect on the FMA-UE score (*F*_[1,161.7]_ = 23.64, *P* = .0001, partial η^2^ = 0.01). The confounder-adjusted estimates revealed that age (*P* = .98), sex (*P* = .53), time from the onset in months (*P* = .46), and dominant hand (*P* = .28) had no major effects on the FMA-UE scores. Compared with the delta value (pre- and post-NEURO), the FMA-UE score showed no difference (*t* = −0.727, *P* = .468, Cohen *d* = 0.035).

**Figure 4:**
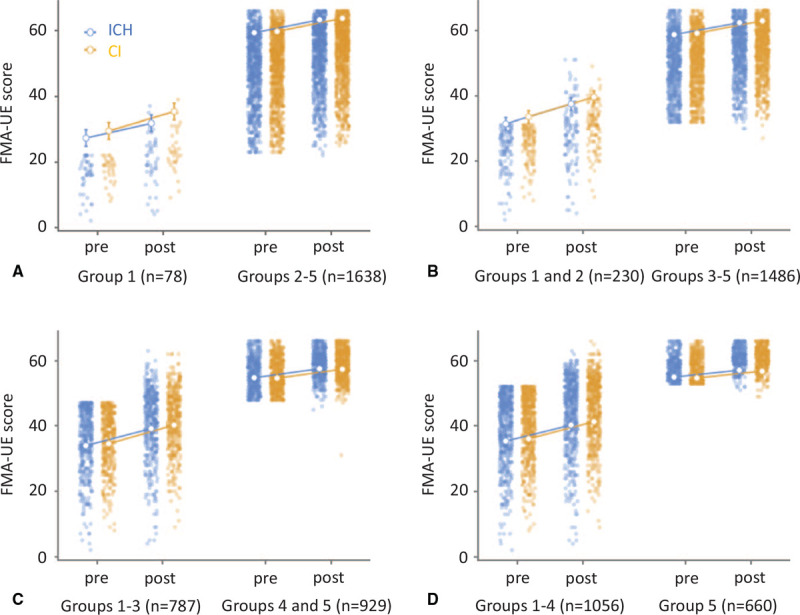
Results of sensitivity analysis compared with the effects of treatment based on FMA scores of the upper extremity in patients with cerebral infarction and intracerebral hemorrhage divided by the pretreatment FMA-UE score: group 1: no (<23), group 2: poor (≤23 to <32), group 3: limited (≤32 to <42), group 4: notable (≤42 to <53), or group 5: full (≤53) recovery capacity. A, Group comparison 1 | 2, 3, 4, 5. B, Group comparison 1, 2 | 3, 4, 5. C, Group comparison 1, 2, 3 | 4, 5. D, Group comparison 1, 2, 3, 4 | 5. No significant interactions were found between diagnosis (cerebral infarction and intracerebral hemorrhage), time series (pre- and post-NEURO), and recovery capacity prediction of FMA-UE (group 1 vs 2–5, *F*_[1,15.6]_ = 1.94, *P* = .16, partial η^2^ = 0.00; group 1, 2 vs 3–5, *F*_[1,0.6]_ =  0.08, *P* = .78, partial η^2^ = 0.0001; group 1–3 vs 4–5: *F*_[1,12.9]_ = 1.81, *P* = .18, partial η^2^ = 0.0001, group 1–4 vs 5: *F*_[1,6.0]_ = 0.87, *P* = .35, partial η^2^ = 0.0001). Open circles and error bars denote the estimated marginal mean and standard errors.

In the sensitivity analysis-stratified analysis, patients were divided into 5 groups based on pre-treatment scores of the FMA-UE for predicting recovery capacity (Table 3). Results of the sensitivity analysis-stratified analysis demonstrated no significant interaction between diagnosis (CI and ICH), time series (pre- and post-NEURO), and recovery capacity prediction of FMA-UE (group 1 vs groups 2–5, *F*_[1,__15.6]_ = 1.94, *P* = .16, partial η^2^ = 0.00; group 1, 2 vs 3–5, *F*_[1,0.6]_ = 0.08, *P* = .78, partial η^2^ = 0.0001; group 1–3 vs 4–5: *F*_[1,12.9]_ = 1.81, *P* = .18, partial η^2^ = 0.0001, group 1–4 vs 5: *F*_[1,6.0]_ = 0.87, *P* = .35, partial η^2^ = 0.0001) (Fig. 4). Post-hoc analysis showed significant changes in FMA-UE scores (median, 25th–75th percentiles) before (49, 38–56 in ICH; 50, 39–56 in CI) and after (53, 43–60 in ICH; 54, 44–60 in CI) NEURO (*P*_Bonferroni_ < .001, Table 3).

**Table 3:** Post-hoc comparisons of the Fugl-Meyer assessment score for upper extremity function scores of the groups according to pretreatment status.

			FMA-UE score		Statistics		
Diagnosis	Group	N	Pre	Post	*t*	*P*_Bonferroni_	Cohen *d*
ICH	1_no	45	17 (14–20)	20 (17–25)	4.86	<.001	1.02
	2_poor	72	27 (25–29)	33 (17–38)	9.43	<.001	1.56
	3_limited	283	41 (36–45)	46 (41–50)	13.06	<.001	1.09
	4_notable	128	50 (49–51)	55 (52–56)	7.75	<.001	0.97
	5_full	312	58 (55–61)	61 (59–63)	6.36	<.001	0.51
	total	840	49 (38–56)	53 (43–60)	16.24	<.001	1.34
CI	1_no	33	19 (16–20)	23 (20–28)	5.48	<.001	1.35
	2_poor	80	28 (25–30)	33 (31–37)	8.81	<.001	1.39
	3_limited	274	41 (37–44)	46 (42–50)	14.91	<.001	1.27
	4_notable	141	50 (49–51)	54 (52–57)	8.21	<.001	0.97
	5_full	348	58 (55–61)	61 (58–63)	6.76	<.001	0.51
	total	876	50 (39–56)	54 (44–60)	16.38	<.001	1.10

All results remained unchanged even when analyzed without imputed data.

## 4 Discussion/conclusion

By analyzing the main effect of NEURO's time series, we confirmed that the upper extremity motor function in stroke patients significantly recovered with NEURO in this study. This result was consistent with those of previous studies.^[15,18,23]^ Furthermore, the major finding of the present study was that no difference in NEURO treatment outcomes was observed with the type of stroke (ICH or CI) even after sensitivity analysis-stratified analysis. These results suggest that long-term upper extremity muscle paralysis can be improved by NEURO equally in patients with CI and ICH.

What is the mechanism of improvement of FMA-UE after 2 weeks of NEURO treatment? In NEURO, the patient actively moves the upper extremity and hand/fingers with OT after rTMS. rTMS is usually applied during the chronic phase of stroke for the treatment of hemiparesis. At high frequency (HF, >5 Hz),^[35]^ rTMS increases the long-term potentiation of neural activity, whereas at low frequency (LF, 1 Hz), it reduces long-term depression.^[36]^ Thus, excitation of the affected cortex with HF-rTMS and inhibition of the unaffected cortex with LF-rTMS is based on the interhemispheric inhibition theory.^[37,38]^ Notably, controlling the excitability of the motor cortex using rTMS while the patient is actively using the upper extremity exemplifies the effectiveness of NEURO treatment. In the chronic phase of stroke, the most widely accepted explanation for the efficacy of the 1-Hz stimulation of the unaffected hemisphere is a reduction in the abnormally high transcallosal inhibition toward the affected hemisphere^[39,40]^; that is, it was suggested that rTMS attenuated brain activation on the intact side and reduced interhemispheric inhibition; conversely, activation on the affected side increased hand mobility in patients, resulting in an improved FMA score.

According to a previous study, the lesion volume was much smaller in ischemic stroke patients than in hemorrhage patients.^[3]^ A large proportion of ICH and CI reported by Edwardson et al. was subcortical,^[3]^ while approximately half of the reported strokes were in the region of the middle cerebral artery in population-based studies. The size- and brain region-related differences are significant factors that influence outcomes (i.e., motor recovery), particularly in the acute to subacute phase. It has been suggested that the type of stroke (i.e., hemorrhage and infarction) does not affect the recovery of upper extremity muscle paralysis even after intensive care,^[3]^ although our study included patients with chronic stroke. The extent of nerve damage affects the recovery of motor paralysis, but there may be no differences in recovery by NEURO in patients with chronic stroke.

Our study has certain limitations. First, we did not analyze the effect of the affected area of the brain on the outcome. Some previous studies have reported that the prediction of motor recovery in patients with stroke-related muscle paralysis in the acute phase depends on the location of the damaged neural area identified by brain imaging.^[41,42]^ In contrast, other studies have reported that recovery can be predicted accurately without brain imaging studies.^[43]^ Therefore, we assume that the location of brain damage affects recovery from motor paralysis in individual patients but not in patients examined during the chronic phase or from the general population. Detailed analysis of the effect of the damaged brain area should be conducted in future studies. Second, our study did not analyze the nutritional status of participating patients. Evidence suggests that nutrition is a key predictor of stroke outcomes.^[44]^ Thus, the nutritional status and its associated impact on NEURO and motor function should be investigated in a separate study. Third, the OT training sessions were not equivalent among patients, although the duration, session time, and frequency were equivalent. Any small discrepancy in OT sessions would interfere with the results. In NEURO OT sessions, the amount of exercise should be measured to determine the outcome of the patient's upper limb exercise therapy or daily life activity. In addition, physical therapy was administered along with OT to some patients. However, we did not specifically investigate the proportion of patients who received physiotherapy. The effect of physiotherapy on motor functional recovery was not analyzed in this study and should be elucidated in future studies.

In conclusion, in the present study, we demonstrated the beneficial clinical effects of intensive NEURO (i.e., rTMS and OT) on upper extremity muscle paralysis and showed that the effects were not influenced by the type of stroke (i.e., ICH or infarction). We recommend NEURO for patients with chronic stroke, including those with CI and hemorrhage.

## Author contributions

**Conceptualization:** Jinichi Sasanuma, Masahiro Abo.

**Data curation:** Hisashi Tatsuno, Jinichi Sasanuma, Kiyohito Kakita, Takatsugu Okamoto, Masato Shimizu.

**Formal analysis:** Hisashi Tatsuno, Toyohiro Hamaguchi, Naoki Nakaya.

**Funding acquisition:** Masahiro Abo.

**Investigation:** Jinichi Sasanuma, Kiyohito Kakita, Takatsugu Okamoto, Masato Shimizu.

**Methodology:** Hisashi Tatsuno, Masato Shimizu, Naoki Nakaya, Masahiro Abo.

**Project administration:** Masahiro Abo.

**Resources:** Kiyohito Kakita, Takatsugu Okamoto.

**Software:** Toyohiro Hamaguchi.

**Supervision:** Jinichi Sasanuma.

**Validation:** Kiyohito Kakita, Takatsugu Okamoto, Masato Shimizu, Masahiro Abo.

**Visualization:** Toyohiro Hamaguchi, Naoki Nakaya.

**Writing – original draft:** Hisashi Tatsuno, Toyohiro Hamaguchi, Masahiro Abo.

**Writing – review & editing:** Toyohiro Hamaguchi, Masahiro Abo.

## References

[1] Andersen KK Olsen TS Dehlendorff C Kammersgaard LP. Hemorrhagic and ischemic strokes compared: stroke severity, mortality, and risk factors. Stroke 2009;40:2068–72. 19359645 10.1161/STROKEAHA.108.540112

[2] Steinke W Sacco RL Mohr JP. Thalamic stroke: presentation and prognosis of infarcts and hemorrhages. Arch Neurol 1992;49:703–10. 1497496 10.1001/archneur.1992.00530310045011

[3] Edwardson MA Wang X Liu B. Stroke lesions in a large upper limb rehabilitation trial cohort rarely match lesions in common preclinical models. Neurorehabil Neural Repair 2017;31:509–20. 28337932 10.1177/1545968316688799 PMC5433918

[4] Murray CJ Lopez AD. Global mortality, disability, and the contribution of risk factors: Global Burden of Disease Study. Lancet 1997;349:1436–42. 9164317 10.1016/S0140-6736(96)07495-8

[5] Rocha S Silva E Foerster A. The impact of transcranial direct current stimulation (tDCS) combined with modified constraint-induced movement therapy (mCIMT) on upper limb function in chronic stroke: a double-blind randomized controlled trial. Disabil Rehabil 2016;38:653–60. 26061222 10.3109/09638288.2015.1055382

[6] Takekawa T Kakuda W Uchiyama M Ikegaya M Abo M. Brain perfusion and upper limb motor function: a pilot study on the correlation between evolution of asymmetry in cerebral blood flow and improvement in Fugl-Meyer Assessment score after rTMS in chronic post-stroke patients. J Neuroradiol 2014;41:177–83. 23886875 10.1016/j.neurad.2013.06.006

[7] Yamada N Kakuda W Senoo A. Functional cortical reorganization after low-frequency repetitive transcranial magnetic stimulation plus intensive occupational therapy for upper limb hemiparesis: evaluation by functional magnetic resonance imaging in poststroke patients. Int J Stroke 2013;8:422–9. 23692672 10.1111/ijs.12056

[8] Kondo T Kakuda W Yamada N Shimizu M Hagino H Abo M. Effect of low-frequency rTMS on motor neuron excitability after stroke. Acta Neurol Scand 2013;127:26–30. 22494271 10.1111/j.1600-0404.2012.01669.x

[9] Kondo T Kakuda W Yamada N Shimizu M Abo M. Effects of repetitive transcranial magnetic stimulation and intensive occupational therapy on motor neuron excitability in poststroke hemiparetic patients: a neurophysiological investigation using F-wave parameters. Int J Neurosci 2015;125:25–31. 24564818 10.3109/00207454.2014.897706

[10] Kakuda W Abo M Kobayashi K. Anti-spastic effect of low-frequency rTMS applied with occupational therapy in post-stroke patients with upper limb hemiparesis. Brain Inj 2011;25:496–502. 21456998 10.3109/02699052.2011.559610

[11] Kakuda W Abo M Kobayashi K. Baseline severity of upper limb hemiparesis influences the outcome of low-frequency rTMS combined with intensive occupational therapy in patients who have had a stroke. PM R 2011;3:516–22. quiz 522. 21665163 10.1016/j.pmrj.2011.02.015

[12] Ueda R Yamada N Abo M Senoo A. White matter changes follow low-frequency repetitive transcranial magnetic stimulation plus intensive occupational therapy for motor paralysis after stroke: a DTI study using TBSS. Acta Neurol Belg 2019;121:387–96. 31115787 10.1007/s13760-019-01150-2

[13] Yuan X Yang Y Cao N Jiang C. Promotion of poststroke motor-function recovery with repetitive transcranial magnetic stimulation by regulating the interhemispheric imbalance. Brain Sci 2020;10: 10.3390/brainsci10090648 PMC7563987 32961836

[14] Ueda R Yamada N Abo M Ruwan PW Senoo A. MRI evaluation of motor function recovery by rTMS and intensive occupational therapy and changes in the activity of motor cortex. Int J Neurosci 2020;130:309–17. 31607202 10.1080/00207454.2019.1680553

[15] Kakuda W Abo M Sasanuma J. Combination protocol of low-frequency rTMS and intensive occupational therapy for post-stroke upper limb hemiparesis: a 6-year experience of more than 1700 Japanese patients. Transl Stroke Res 2016;7:172–9. 26884316 10.1007/s12975-016-0456-8

[16] Rossi S Hallett M Rossini PM Pascual-Leone A. Safety of TMS Consensus Group. Safety of TMS Consensus Group. Safety, ethical considerations, and application guidelines for the use of transcranial magnetic stimulation in clinical practice and research. Clin Neurophysiol 2009;120:2008–39. 19833552 10.1016/j.clinph.2009.08.016 PMC3260536

[17] Tamashiro H Kinoshita S Okamoto T Urushidani N Abo M. Effect of baseline brain activity on response to low-frequency rTMS/intensive occupational therapy in poststroke patients with upper limb hemiparesis: a near-infrared spectroscopy study. Int J Neurosci 2018;129:337–43. 30311827 10.1080/00207454.2018.1536053

[18] Urushidani N Okamoto T Kinoshita S. Combination treatment of low-frequency repetitive transcranial magnetic stimulation and intensive occupational therapy for ataxic hemiparesis due to thalamic hemorrhage. Case Rep Neurol 2017;9:179–87. 28966585 10.1159/000478975 PMC5618400

[19] Du J Yang F Hu J. Effects of high- and low-frequency repetitive transcranial magnetic stimulation on motor recovery in early stroke patients: evidence from a randomized controlled trial with clinical, neurophysiological and functional imaging assessments. Neuroimage Clin 2019;21:101620. 30527907 10.1016/j.nicl.2018.101620 PMC6411653

[20] Watanabe K Kudo Y Sugawara E. Comparative study of ipsilesional and contralesional repetitive transcranial magnetic stimulations for acute infarction. J Neurol Sci 2018;384:10–4. 29249365 10.1016/j.jns.2017.11.001

[21] Wolf SL Winstein CJ Miller JP. Effect of constraint-induced movement therapy on upper extremity function 3 to 9 months after stroke: the EXCITE randomized clinical trial. JAMA 2006;296:2095–104. 17077374 10.1001/jama.296.17.2095

[22] Avenanti A Coccia M Ladavas E Provinciali L Ceravolo MG. Low-frequency rTMS promotes use-dependent motor plasticity in chronic stroke: a randomized trial. Neurology 2012;78:256–64. 22238412 10.1212/WNL.0b013e3182436558

[23] Abo M Kakuda W Momosaki R. Randomized, multicenter, comparative study of NEURO versus CIMT in poststroke patients with upper limb hemiparesis: the NEURO-VERIFY Study. Int J Stroke 2014;9:607–12. 24015934 10.1111/ijs.12100

[24] Kakuda W Abo M Shimizu M. A multi-center study on low-frequency rTMS combined with intensive occupational therapy for upper limb hemiparesis in post-stroke patients. J Neuroeng Rehabil 2012;9:04. 10.1186/1743-0003-9-4 PMC3271959 22264239

[25] Gladstone DJ Danells CJ Black SE. The Fugl-Meyer assessment of motor recovery after stroke: a critical review of its measurement properties. Neurorehabil Neural Repair 2002;16:232–40. 12234086 10.1177/154596802401105171

[26] Hoonhorst MH Nijland RH Van den Berg JS Emmelot CH Kollen BJ Kwakkel G. How do Fugl-Meyer arm motor scores relate to dexterity according to the Action Research Arm Test at 6 months poststroke? Arch Phys Med Rehabil 2015;96:1845–9. 26143054 10.1016/j.apmr.2015.06.009

[27] Lundquist CB Maribo T. The Fugl-Meyer assessment of the upper extremity: reliability, responsiveness and validity of the Danish version. Disabil Rehabil 2017;39:934–9. 27062881 10.3109/09638288.2016.1163422

[28] Tripepi G Chesnaye NC Dekker FW Zoccali C Jager KJ. Intention to treat and per protocol analysis in clinical trials. Nephrology (Carlton) 2020;25:513–7. 32147926 10.1111/nep.13709

[29] Del Re AC Maisel NC Blodgett JC Finney JW. Intention-to-treat analyses and missing data approaches in pharmacotherapy trials for alcohol use disorders. BMJ Open 2013;3:e003464. 10.1136/bmjopen-2013-003464 PMC3831108 24227870

[30] Han CE Arbib MA Schweighofer N. Stroke rehabilitation reaches a threshold. PLoS Comput Biol 2008;4:e1000133. 18769588 10.1371/journal.pcbi.1000133 PMC2527783

[31] Kim TH Vemuganti R. Effect of sex and age interactions on functional outcome after stroke. CNS Neurosci Ther 2015;21:327–36. 25404174 10.1111/cns.12346 PMC6495347

[32] Vincent-Onabajo GO Hamzat TK Owolabi MO. Are there gender differences in longitudinal patterns of functioning in Nigerian stroke survivors during the first year after stroke? NeuroRehabilitation 2014;34:297–304. 24448876 10.3233/NRE-141047

[33] Beuscher VD Kuramatsu JB Gerner ST. Functional long-term outcome after left- versus right-sided intracerebral hemorrhage. Cerebrovasc Dis 2017;43:117–23. 28049189 10.1159/000454775

[34] Luft AR Waller S Forrester L. Lesion location alters brain activation in chronically impaired stroke survivors. Neuroimage 2004;21:924–35. 15006659 10.1016/j.neuroimage.2003.10.026

[35] Maeda F Keenan JP Tormos JM Topka H Pascual-Leone A. Modulation of corticospinal excitability by repetitive transcranial magnetic stimulation. Clin Neurophysiol 2000;111:800–5. 10802449 10.1016/s1388-2457(99)00323-5

[36] Chen R Classen J Gerloff C. Depression of motor cortex excitability by low-frequency transcranial magnetic stimulation. Neurology 1997;48:1398–403. 9153480 10.1212/wnl.48.5.1398

[37] Bertolucci F Chisari C Fregni F. The potential dual role of transcallosal inhibition in post-stroke motor recovery. Restor Neurol Neurosci 2018;36:83–97. 29439366 10.3233/RNN-170778

[38] Wang RY Wang FY Huang SF Yan YR. High-frequency repetitive transcranial magnetic stimulation enhanced treadmill training effects on gait performance in individuals with chronic stroke: a double-blinded randomized controlled pilot trial. Gait Posture 2019;68:382–7. 30586670 10.1016/j.gaitpost.2018.12.023

[39] Xu AH Sun YX. Research hotspots and effectiveness of repetitive transcranial magnetic stimulation in stroke rehabilitation. Neural Regen Res 2020;15:2089–97. 32394967 10.4103/1673-5374.282269 PMC7716019

[40] McDonnell MN Stinear CM. TMS measures of motor cortex function after stroke: a meta-analysis. Brain Stimul 2017;10:721–34. 28385535 10.1016/j.brs.2017.03.008

[41] Prabhakaran S Zarahn E Riley C. Inter-individual variability in the capacity for motor recovery after ischemic stroke. Neurorehabil Neural Repair 2008;22:64–71. 17687024 10.1177/1545968307305302

[42] Stinear CM Barber PA Petoe M Anwar S Byblow WD. The PREP algorithm predicts potential for upper limb recovery after stroke. Brain 2012;135 (pt 8):2527–35. 22689909 10.1093/brain/aws146

[43] Stinear CM Byblow WD Ackerley SJ Smith MC Borges VM Barber PA. PREP2: A biomarker-based algorithm for predicting upper limb function after stroke. Ann Clin Transl Neurol 2017;4:811–20. 29159193 10.1002/acn3.488 PMC5682112

[44] Samavarchi Tehrani S Khatami SH Saadat P. Association of serum magnesium levels with risk factors, severity and prognosis in ischemic and hemorrhagic stroke patients. Caspian J Intern Med 2020;11:83–91. 32042391 10.22088/cjim.11.1.83 PMC6992732

